# Draft Genome Sequence of *Porphyrobacter mercurialis* (sp. nov.) Strain Coronado

**DOI:** 10.1128/genomeA.00856-15

**Published:** 2015-11-19

**Authors:** David A. Coil, Jonathan A. Eisen

**Affiliations:** a University of California Davis Genome Center, Davis, California, USA; b Department of Evolution and Ecology, University of California Davis, Davis, California, USA; c Department of Medical Microbiology and Immunology, University of California Davis, Davis, California, USA

## Abstract

Here, we present the draft genome of *Porphyrobacter mercurialis* strain Coronado, the proposed type strain for this species. The assembly contains 3,482,341 bp in 10 contigs.

## GENOME ANNOUNCEMENT

As part of a nationwide citizen science project, Project MERCCURI (spacemicrobes.org), swabs were collected from a variety of high-touch surfaces around the United States. In one such sampling, *Porphyrobacter mercurialis* strain Coronado was collected from a stadium seat at Niedermeyer Field, Coronado High School, in Coronado, CA. Swabs were plated onto lysogeny broth (LB) agar plates and then double dilution streaked. Genomic DNA was extracted using a Wizard Genomic DNA purification kit (Promega) from fresh overnight cultures. Illumina paired-end libraries were then made from sonicated DNA using a TruSeq DNA sample prep kit version 2 (Illumina).

A total of 5,043,828 paired-end reads were generated on an Illumina MiSeq platform, at a read length of 300 bp. Quality trimming and error correction resulted in 4,632,516 high-quality reads. All sequence processing and assembly were performed using the A5 assembly pipeline (1). The assembly produced 10 contigs, (*N*_50_ = 2,089,745), totaling 3,482,341 bp, with a GC content of 67% and an estimated overall coverage of 280×. Completeness of the genome was assessed using PhyloSift (2), which searches for 37 highly conserved, single copy marker genes (3), all of which were found in this assembly.

Automated annotation was performed using the RAST server (4). *P. mercurialis* strain Coronado contains 3,158 predicted protein-coding sequences and 51 predicted noncoding RNAs. A full-length (1,482 bp) 16S sequence was obtained from this annotation and was used to attempt to identify the isolate. The isolate clearly fell within the *Erythrobacteraceae* family (5) but did not group with any known genera and was only 95% similar to the closest cultured match, *Porphyrobacter sanguineus* (6). Therefore, we have formally characterized this species and are simultaneously submitting the genome announcement and a species description (D. Coil, J. Flanagan, A. Stump, A. Alexiev, J. Lang, and J. Eisen, “*Porphyrobacter mercurialis* sp. nov., isolated from a stadium seat and emended description of the genus *Porphyrobacter*,” submitted for publication).

### Nucleotide sequence accession numbers.

This whole-genome shotgun project has been deposited at DDBJ/EMBL/GenBank under the accession number JTDN00000000. The version described in this paper is the first version, JTDN01000000. The raw Illumina reads are available at ENA/SRA under the accession number PRJEB8004.
